# Case Report: A New Subtype of Lynch Syndrome Associated With *MSH2* c.1024_1026 Identified in a Chinese Family

**DOI:** 10.3389/fmed.2022.811368

**Published:** 2022-01-28

**Authors:** Lu Li, Zhe Zhao, Lin Dong, Jia Jia, Ke Su, Hua Bai, Jie Wang

**Affiliations:** ^1^Health Service Department of the Guard Bureau of the Joint Staff Department, Beijing, China; ^2^Department of Medical Oncology, Beijing Hospital, National Center of Gerontology, Institute of Geriatric Medicine, Chinese Academy of Medical Sciences, Beijing, China; ^3^Department of Medical Oncology, National Cancer Center/National Clinical Research Center for Cancer/Cancer Hospital, Chinese Academy of Medical Sciences and Peking Union Medical College, Beijing, China; ^4^Department of Pathology, National Cancer Center/National Clinical Research Center for Cancer/Cancer Hospital, Chinese Academy of Medical Sciences and Peking Union Medical College, Beijing, China; ^5^Genetron Health (Beijing) Co., Ltd., Beijing, China

**Keywords:** DNA mismatch repair, hereditary nonpolyposis colorectal neoplasms, immunotherapy, MutS homolog 2 protein, skin neoplasms, case report

## Abstract

**Background:**

Lynch syndrome is an autosomal dominant disorder associated with a high incidence of various cancer types. Multiple variants of mismatch repair genes have been reported for Lynch syndrome. However, the diagnosis in patients with atypical cancer types remains challenging. Specifically, little is known about the genetic background of Lynch syndrome-related renal carcinoma. We present a case wherein a renal carcinoma patient with multiple primary skin tumors harbored a variant that has not been previously shown to be associated with Lynch syndrome.

**Case Presentation:**

The proband was a 60-year-old Chinese man with a history of Lynch syndrome-related renal carcinoma and recurrent primary skin tumors. Immunohistochemistry revealed loss of MSH2 and MSH6. Sequencing of mismatch repair genes revealed a previously unknown germline *MSH2* mutation (c.1024_1026), which results in an amino acid deletion (p.V342). This variant was co-segregated among the carcinoma-affected family members. After six cycles of immunotherapy, a marked regression of the skin tumors was observed.

**Conclusions:**

We clarify the pathogenic significance of this newly described mutation and suggest immunotherapy for patients with this subtype of Lynch syndrome.

## Introduction

Lynch syndrome (LS), previously known as hereditary nonpolyposis colorectal cancer, is an autosomal dominant disorder that is associated with a high risk of developing colorectal and other cancers (1, 2). The pathogenesis of LS is associated with mutations in DNA mismatch repair (MMR) genes, including those in *MLH1, MSH2, MSH6, PMS2*, and *EPCAM* (3, 4). These mutations cause deficiencies in DNA repair, resulting in a high frequency of replication errors. Genetic analyses and protein expression analysis using immunohistochemistry (IHC) are essential for diagnosing LS (5, 6).

Approximately 1 in 350 individuals has LS (7), and different subtypes are associated with varying clinical symptoms and pathological features. Of the individuals with LS, 9.2% are affected by Muir-Torre syndrome (MTS) (8). MTS is primarily related to *MSH2* mutations and is characterized by skin tumors (such as sebaceous adenomas and skin squamous cell carcinomas) (9). However, MTS-related primary renal carcinomas with skin tumors have rarely been reported. We report a case of LS originating as a renal cell carcinoma with multiple primary tumors and a previously unknown *MSH2* mutation (c.1024_1026).

## Case Description

**Figure 1:**
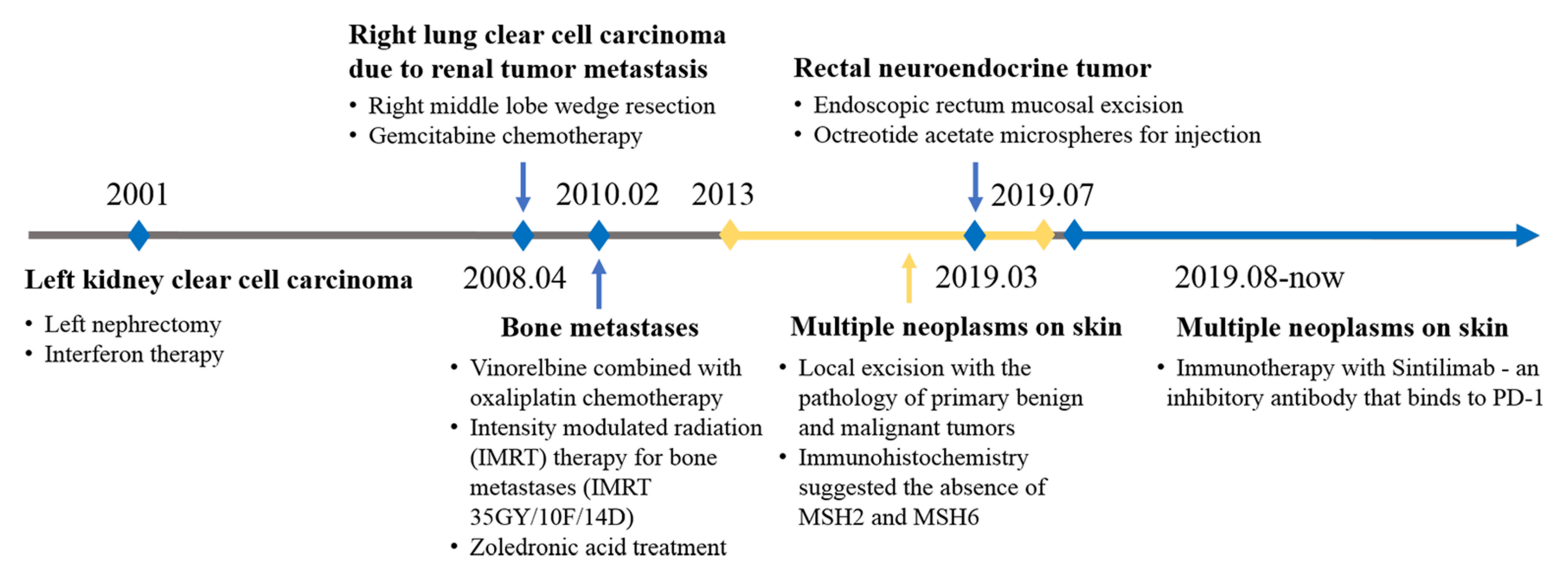
Timeline of disease diagnosis and treatment.

The proband had a history of various cancers (Figure 1). In 2001, at the age of 41 years, he was diagnosed with left kidney clear cell carcinoma. In 2008, he was diagnosed with right lung clear cell carcinoma due to renal tumor metastasis, in 2010 with bone metastases, and in 2019, he was diagnosed with a rectal submucosal neuroendocrine tumor. The proband received the standard treatment for each cancer.

**Figure 2:**
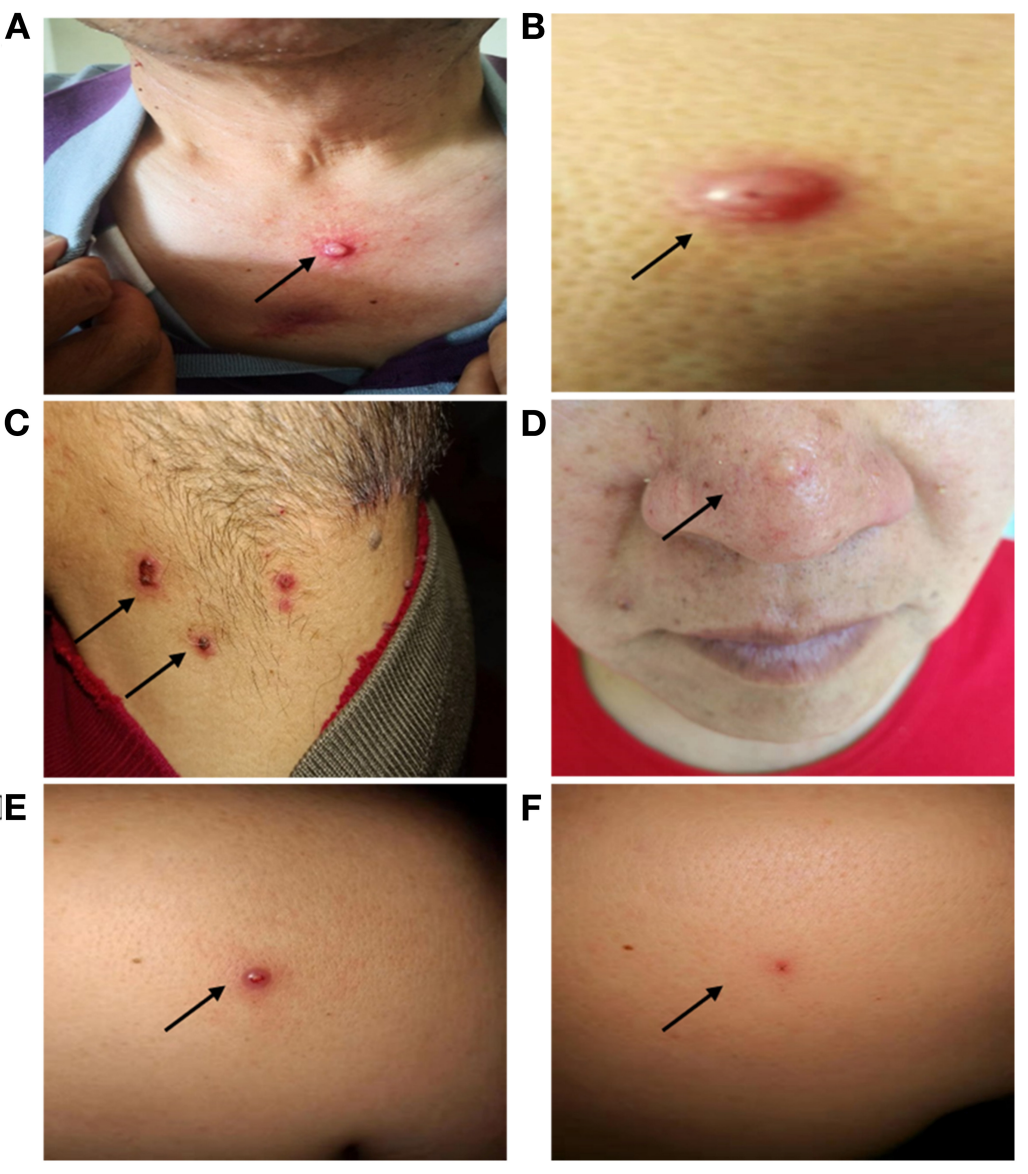
Clinical manifestations of the proband's skin tumors. The proband presented with recurrent skin tumors on the chest (squamous cell carcinoma) **(A)**, back (hyperplastic squamous cell carcinoma) **(B)**, neck (sebaceous adenoma) **(C)**, face (sebaceous adenoma) **(D)**. A nodule in the back shoulder before (0.9 × 0.6 cm) **(E)**, and after (0.2 × 0.1 cm) **(F)** immunotherapy.

In addition to the internal carcinomas, the proband also presented with multiple neoplasms, which developed sequentially, located on the skin of the head and neck or upper chest, with faster growth starting in 2013 (Figure 2). Pathology revealed primary benign and malignant tumors, including skin squamous cell carcinoma, keratoacanthoma, sebaceous adenoma, and sebaceous gland carcinoma (Supplementary Table 1). Starting in May 2019, the proband exhibited recurring skin tumors, at a rate of 15–17 per month. The tumors were removed and diagnosed as primary skin malignant tumors.

### Family History

One brother of the proband died of gastric cancer and three sisters were all diagnosed with endometrial cancer, with one case accompanied by metachronous rectal cancer. Only one brother was cancer-free. In addition, a 47-year-old daughter of one of his sisters was also diagnosed with endometrial cancer. Because of the family history of LS-like cancers and skin lesions, the MTS subtype was considered as a diagnosis. Comprehensive genetic testing was performed, and genetic counseling was offered.

### Laboratory Tests

**Figure 3:**
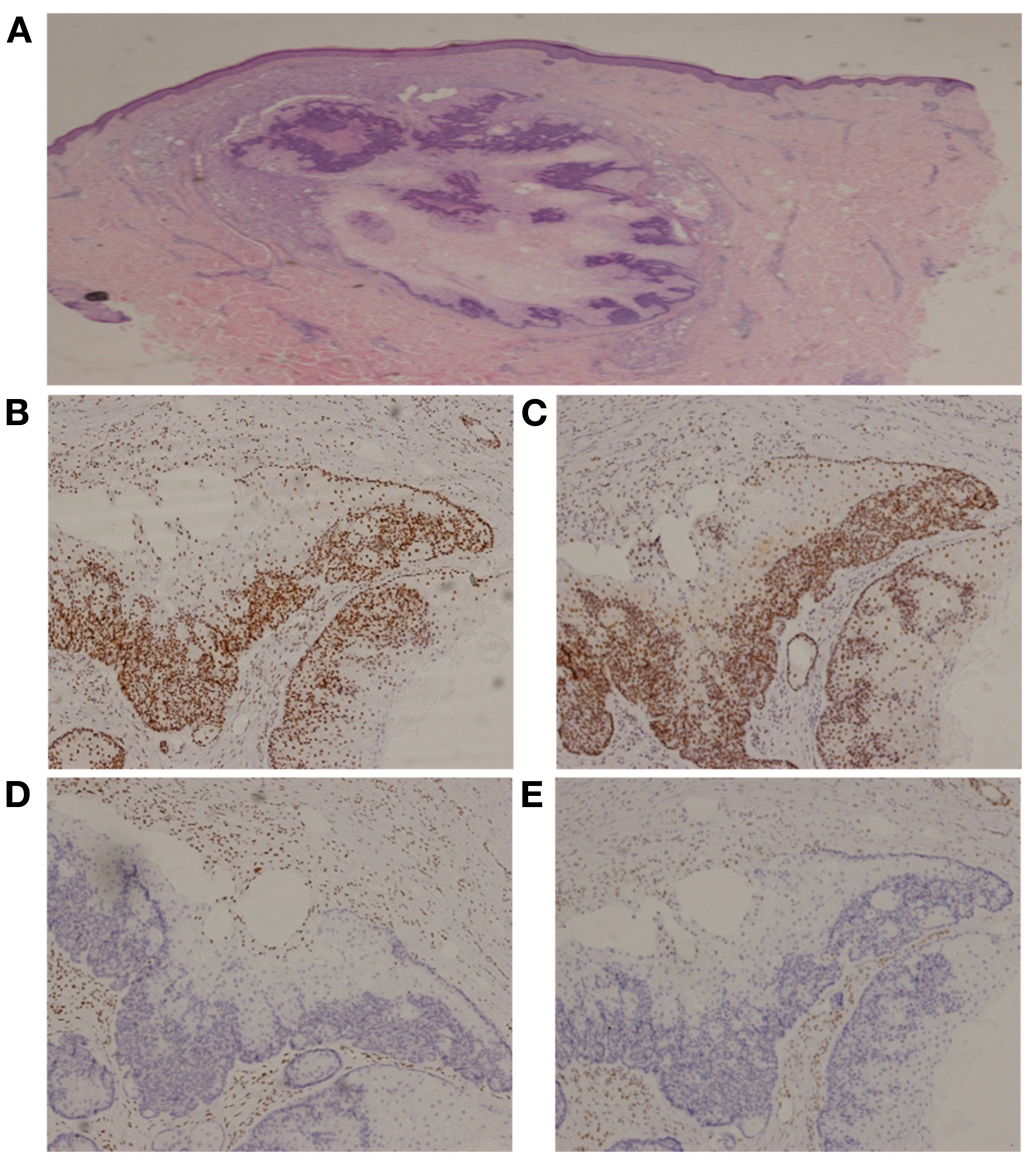
Immunohistochemistry of a sebaceous gland adenoma. **(A)** Hematoxylin and Eosin, 20x; **(B)** MLH1, 100x; **(C)** PMS2, 100x; **(D)** MSH2, 100x; **(E)** MSH6, 100x.

MMR expression in the visceral and reduplicative skin neoplasms of the proband (Supplementary Table 2) was assessed using IHC with MaxVisionTM primary antibodies (anti-MLH1, MAB-0789; anti-PMS2, RMA-0775; anti-MSH2, MAB-0836; anti-MSH6, RMA-0770); results revealed the absence of MSH2 and MSH6 expression (Figure 3). Microsatellite instability (MSI) analysis of the genomic DNA—using a 5-mononucleotide marker panel (BAT-25, BAT-26, NR-21, NR-24, and MONO-27)—and PCR analysis—using capillary electrophoresis on an ABI 3130xl Genetic Analyzer (Applied Biosystems, Forster City, CA)—generated inconsistent results, with MSI-high status for the chest squamous cell carcinoma (Supplementary Table 2). Whole exome sequencing on multiple tumor tissues and peripheral blood—performed using next-generation sequencing (Supplementary Table 2)—revealed a germline heterozygous deletion (c.1024_1026) in exon 6 of *MSH2* on chromosome 2, resulting in a deletion of the valine residue at position 342 (p.V342). This autosomal dominant mutation excludes promoter methylation and has not been reported previously.

**Figure 4:**
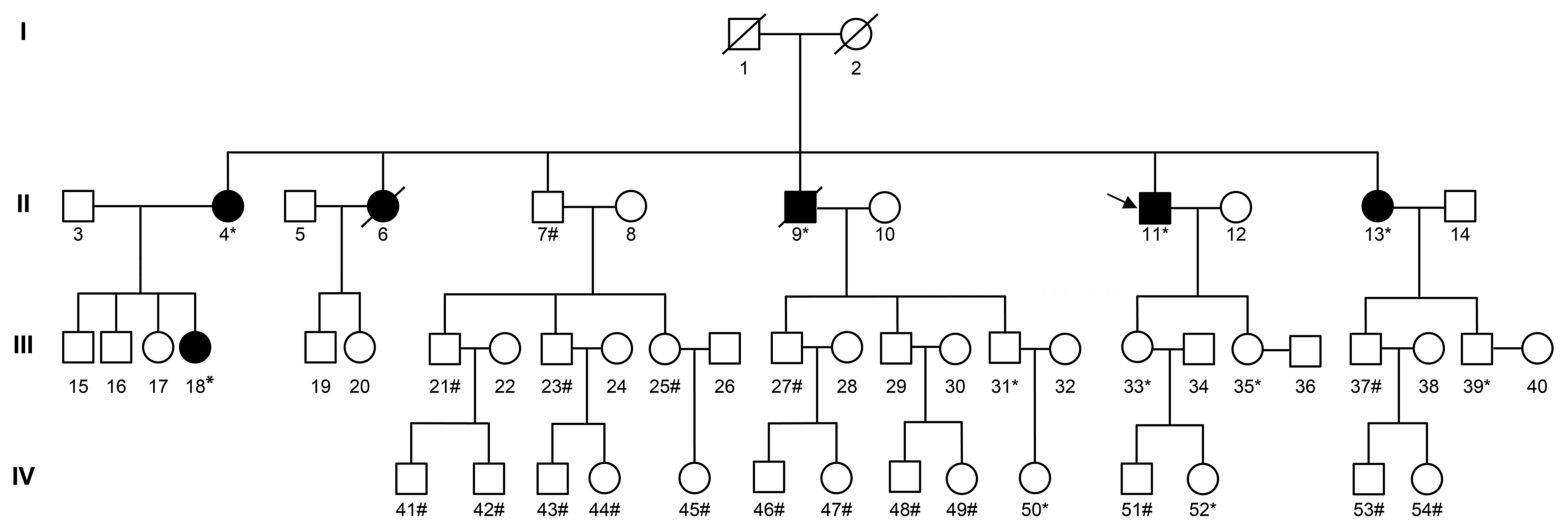
Pedigree of the family with *MSH2* c.1024_1026. The arrow (→) indicates the proband. Squares and circles denote males and females, respectively. Roman numerals indicate generations. Black solids represent tumor patients. Patients number 4, 6, 13, and 18 suffered from endometrial carcinoma before the age of 50. Furthermore, patient number 4 also suffered from rectal carcinoma at the age of 52. Patient number 9 suffered from gastric carcinoma at the age of 60. Patient number 11 suffered from renal carcinoma at the age of 41, skin carcinoma at the age of 53, and a rectal neuroendocrine tumor at the age of 58. The asterisk (*) indicates *MSH2* c.1024_1026 carriers. The pound (#) indicates non-carriers. The remaining family members have not been tested for *MSH2* mutations.

IHC on the tumor tissues obtained from the siblings and niece of the proband also revealed absence of MSH2 and MSH6 expression, along with microsatellite-stable status (Supplementary Table 3). The same *MSH2* mutation (c.1024_1026, p.V342) was detected in samples from the affected family members, but not in those from the cancer-free sibling (Supplementary Table 3), supporting the pathogenic role of this *MSH2* variant. Further analysis showed that three consecutive generations had inherited the mutation (Figure 4).

## Diagnostic Assessment

### Clinical Diagnosis

Consideration of the multi-primary cancers, family history, and genetic testing resulted in the diagnosis of LS subtype MTS, based on the Amsterdam II criteria (10). The *MSH2* mutation (c.1024_1026) will be documented in genetic databases such as The International Society for Gastrointestinal Hereditary Tumors (InSiGHT).

### Therapeutic Intervention and Outcome

Based on the MMR gene defects and the MSI-high status, the proband was treated with sintilimab—an inhibitory antibody that binds to PD-1—at a dose of 200 mg per 3-week cycle. After 6 cycles, no new skin tumors were observed for nearly 5 months, and the size of the largest skin tumor decreased from 0.9 × 0.6 cm to 0.2 × 0.1 cm (Figure 2). So far, the proband has been treated with immunotherapy for more than a year without new lesions or severe adverse events. The proband's condition is stable and the efficacy of immunotherapy is being monitored.

## Discussion

The presence of multiple sebaceous glands is a sign of MMR gene defects and may serve as a useful clinical parameter for diagnosing the MTS subtype of LS (11). Approximately 70–75% of the LS cases are caused by mutations in *MSH2* and *MLH1* (12). The in-frame deletion of *MSH2* identified in this study (c.1024_1026) has not been reported previously. We have proposed a correlation between *MSH2* and LS/MTS, which clarifies the prognosis of the proband, and provided guidance for early cancer screening in members of the proband's family. Future developments in *in silico* analyses may facilitate improved screening, diagnosis, and prevention.

Irradiation and excision of skin tumors and the use of isotretinoin to prevent recurrence—in conjunction with early detection of visceral tumors—may serve as beneficial strategies for LS/MTS treatment (13, 14). Immunotherapy in LS is mostly used for the treatment of multiple visceral metastases (15), and there is lack of evidence regarding the treatment of repeated skin tumors. Our results suggest that LS/MTS patients with difficult-to-control skin tumors can benefit from immunotherapy, and it is possible that this treatment has a preventive effect on the progress of subsequent visceral cancers. This poses a challenge with respect to monitoring the effectiveness of immunotherapy. Further studies are needed to provide more conclusive evidence.

In conclusion, we propose that the in-frame deletion of *MSH2* (c.1024_1026) is a new pathogenic mutation associated with LS/MTS and that immunotherapy should be considered as a treatment for patients with this subtype of LS.

## Patient Perspective

‘I was extremely troubled by the lumps on my skin, of which the incidence and the frequency of excision were getting higher and higher, before receiving immunotherapy. But after the application of immunotherapy, my skin tumors grew slowly, and the internal carcinomas were maintained stable. I was very satisfied with this. The care and attention I received in the cancer hospital from doctors and nurses was well-organized, comfortable and excellent. I believe I will be better and I also wish other cancer patients progressing.'

## Data Availability Statement

The original contributions presented in the study are included in the article/Supplementary Material, further inquiries can be directed to the corresponding authors.

## Ethics Statement

The studies involving human participants were reviewed and approved by the Human Laboratory Responsibility Committee of the Cancer Hospital of the Chinese Academy of Medical Sciences (Beijing, China). Written informed consent was obtained from the individual(s) for the publication of any potentially identifiable images or data included in this article.

## Author Contributions

LL was responsible for sample acquisition and data interpretation and analysis. ZZ and HB were responsible for the clinical management of the patient and acquisition of data and helped draft the manuscript. LL and ZZ were the major contributors in writing the manuscript. LD and JJ were responsible for the pathological diagnosis and immunohistochemistry. KS was responsible for the genetic testing. JW was responsible for the clinical management, comprehensive interpretation of the data, and scientific revision. All authors have read and approved the final manuscript.

## Funding

This work was supported by National Key Research and Development project (2019YFC1315700); the National Natural Sciences Foundation Key Program [81630071]; CAMS Innovation Fund for Medical Sciences (CIFMS 2016-I2M-3-008); Aiyou foundation (KY201701); Ministry of Education Innovation Team development project (IRT-17R10); and CAMS Key lab of translational research on lung cancer (2018PT31035).

## Conflict of Interest

Author KS was employed by the company Genetron Health. Sequencing was conducted through Genetron Health, and they provided us with interpretations of the sequencing methodology. The remaining authors declare that the research was conducted in the absence of any commercial or financial relationships that could be construed as a potential conflict of interest.

## Publisher's Note

All claims expressed in this article are solely those of the authors and do not necessarily represent those of their affiliated organizations, or those of the publisher, the editors and the reviewers. Any product that may be evaluated in this article, or claim that may be made by its manufacturer, is not guaranteed or endorsed by the publisher.

## Abbreviations

LS: Lynch Syndrome
MTS: Muir-Torre syndrome
MSI, microsatellite instability, MMR: mismatch repair
IHC, immunohistochemistry, TMB: tumor mutational burden.
