# Supplementary material for: Case Report: A New Subtype of Lynch Syndrome Associated With MSH2 c.1024_1026 Identified in a Chinese Family
Source: Front Med (Lausanne). 2022 Jan 28;9:811368. doi: 10.3389/fmed.2022.811368 (PMC8833100; doi:10.3389/fmed.2022.811368)
Supplement: Supplementary file 1 [file Data_Sheet_1.docx]

Supplementary Material

# Supplementary Table 1. Skin lesions of the proband: histology, age at the time of diagnosis, and tumor location.

| **Date (****Age) of Diagnosis** | **Location** | **Histology Pathology** |
| --- | --- | --- |
| September 2013 (53) | Right mouth corner | Hyperplastic squamous cell carcinoma |
| September 2013 (53) | Neck bend on the right | Skin appendage tumor |
| September 2013 (53) | Left eye corner | Hair follicle source tumor |
| November 2013 (53) | Forehead | Keratoacanthoma |
| February 2014 (54) | Left side face | Hyperplastic squamous cell carcinoma |
| October 2014 (54) | Back | Subcutaneous fibrous tissue hyperplasia |
| January 2015 (55) | Nasal side | Inflammation |
| March 2016 (56) | Front chest | Sebaceous carcinoma |
| May 2018 (58)* | Back | Sebaceous adenoma |
| October 2018 (58)* | Chest | Neutrophil squamous cell carcinoma |
| October 2018 (58)* | Back | Hyperplastic squamous cell carcinoma |
| November 2018 (58)* | Scalp | Benign tumors from skin appendages |
| March 2019 (59)  July 2019 (59)* | Neck and scalp  Chest | Sebaceous adenoma  Hyperplastic squamous cell carcinoma |

# Notes: asterisks (*) indicate the tumors used for immunohistochemistry, microsatellite instability, tumor mutational burden, and PD-L1 analysis.

# Supplementary Table 2. Analysis of the patient’s IHC of MMR proteins, microsatellite status, TMB, and PD-L1.

| **Characteristic** | **Time** | **Location** | **Histology** | **IHC** | **Gene** | **Microsatellite status** | **TMB**  **(cut-off)** | **PD-L1** |
| --- | --- | --- | --- | --- | --- | --- | --- | --- |
| Visceral malignancy | April 2008 | Lung | Metastatic clear cell carcinoma | MSH2 (-) MSH6 (-) | MSH2  (c.1024_1026 del) | MSS | 3.09/Mb (20) | TPS <1% |
| Skin benign tumor | May 2018 | Back | Sebaceous adenoma | MSH2 (-) MSH6 (-) | MSH2  (c.1024_1026 del) | MSI-L | Not tested | Not tested |
| Skin malignant tumor | October 2018 | Chest | Squamous cell carcinoma | MSH2 (-) MSH6(-) | MSH2  (c.1024_1026 del) | MSS | 0.58/Mb (20) | negative |
| Benign tumor of skin | November 2018 | Scalp | Benign tumors of skin appendages | MSH2 (-) MSH6 (-) | MSH2  (c.1024_1026 del) | MSS | 9.53/Mb (20) | Not tested |
| Blood | November 2018 | Blood | White blood cell | NT | MSH2  (c.1024_1026 del) | NT | 0/Mb (20) | NT |
| Skin malignant tumor | July 2019 | Chest | Squamous cell carcinoma | MSH2 (-) MSH6 (-) | MSH2  (c.1024_1026 del) | MSI-H | 18.42/Mb (20) | TPS <1% |

# Notes: IHC: immunohistochemistry; MMR: mismatch repair; MSS: microsatellite stable; MSI: microsatellite instable; NT: specimens were not suitable for the corresponding tests; PD-L1: programmed death ligand-1; TMB: tumor mutational burden.

# Supplementary Table 3. Medical history, genetic status, and microsatellite status of the family members of the proband.

| **Family members** | **History (age at diagnosis)** | **Gene** | **Microsatellite status** |
| --- | --- | --- | --- |
| Elder sister (No.4 in Figure 3) | Endometrial carcinoma (50) | MSH2(c.1024_1026 del) | NT |
| Elder sister (No.4 in Figure 3) | Rectal carcinoma (52) | MSH2(c.1024_1026 del) | NT |
| Second sister (No.6 in Figure 3) | Endometrial carcinoma (44) | Not tested because of death | NT |
| Elder brother (No.7 in Figure 3) | No history of cancer | Without mismatch repair gene mutation | NT |
| Second brother (No.9 in Figure 3) | Gastric carcinoma (60) | MSH2(c.1024_1026 del) | MSS |
| The proband (No.11 in Figure 3) | Renal carcinoma (41) | MSH2(c.1024_1026 del) | MSS |
| The proband (No.11 in Figure 3) | Skin tumors (53) | MSH2(c.1024_1026 del) | MSS, MSI-L, or MSI-H |
| Third sister (No.13 in Figure 3) | Endometrial carcinoma (48) | MSH2(c.1024_1026 del) | MSS |
| One daughter of elder sister (No.18 in Figure 3) | Endometrial carcinoma (47) | MSH2(c.1024_1026 del) | MSS |

Notes: MSS: microsatellite stable; MSI: microsatellite instable; NT: specimens were not suitable for the corresponding tests.
